# Treatment strategy and post‐treatment management of colorectal neuroendocrine tumor

**DOI:** 10.1002/deo2.254

**Published:** 2023-06-12

**Authors:** Masau Sekiguchi, Takahisa Matsuda, Yutaka Saito

**Affiliations:** ^1^ Cancer Screening Center National Cancer Center Hospital Tokyo Japan; ^2^ Endoscopy Division National Cancer Center Hospital Tokyo Japan; ^3^ Division of Screening Technology National Cancer Center Institute for Cancer Control Tokyo Japan; ^4^ Division of Gastroenterology and Hepatology Toho University Omori Medical Center Tokyo Japan

**Keywords:** colorectal neuroendocrine tumors, endoscopic resection, lymphovascular invasion, metastasis, radical surgery

## Abstract

Following the increase in colorectal neuroendocrine tumors (NETs), there is a consequent increase in the importance of their appropriate treatment and post‐treatment management. It is widely accepted that colorectal NETs sized ≥20 mm and those with muscularis propria invasion are indicated for radical surgery, and those sized <10 mm without the invasion are indicated for local resection. No consensus has been reached regarding the treatment strategy for those sized 10–19 mm without the invasion. Endoscopic resection has become a primary option for the local resection of colorectal NETs. For rectal NETs sized <10 mm, modified endoscopic mucosal resection, such as endoscopic submucosal resection with ligation device and endoscopic mucosal resection with a cap‐fitted panendoscope, seems favorable because of its ability to achieve a high R0 resection rate, safety, and convenience. Endoscopic submucosal dissection can also be helpful for these lesions; however, this procedure may be more effective for large lesions or those in the colon. Management following local resection of colorectal NETs is based on the pathological evaluation of factors associated with metastasis, including tumor size, invasion depth, tumor cell proliferative activity (NET grading), presence of lymphovascular invasion, and resection margins. There remain unclear issues in managing cases with NET grading ≥2, positive lymphovascular invasion, and positive resection margins following local resection. In particular, there is confusion regarding managing positive lymphovascular invasion because positivity has become remarkably high with the increased use of the immunohistochemical/special staining. Further evidence based on long‐term clinical outcomes is required to address these issues.

## INTRODUCTION

Gastrointestinal (GI) neuroendocrine tumors (NETs) are rare tumors that account for a small portion of GI neoplasia.^1^ However, their prevalence and incidence have reportedly increased worldwide, including in Japan.^2^, ^3^ The Japanese nationwide survey demonstrated that the prevalence of hindgut NETs, including colorectal NETs, the most frequently observed GI‐NETs in Japan, increased from 2.07 per 100,000 population (95% confidence interval [CI] 1.56–2.55) in 2005 to 4.52 (95% CI 3.17–5.87) per 100,000 population in 2010.^3^ This increase is considered to have been influenced by the improvement in colonoscopy imaging and the increasing awareness and access to colorectal cancer screening and colonoscopy. Furthermore, its current true prevalence may be much higher than estimated, considering the difficulty in detecting colorectal NETs and the potential existence of undetected lesions. With the increase in colorectal NETs, the necessity and importance of understanding the appropriate treatment and post‐treatment management have increased. In this article, we review these issues with an emphasis on endoscopic resection and discuss the problems to be solved in the future.

## SELECTION OF TREATMENT FOR COLORECTAL NET

There are several guidelines for the treatment of colorectal NETs worldwide, including the European Neuroendocrine Tumor Society consensus guidelines, National Comprehensive Cancer Network guidelines, North American Neuroendocrine Tumor Society consensus guidelines, and Japanese Neuroendocrine Tumor Society clinical practice guidelines.^4^, ^5^, ^6^, ^7^ A consensus has been reached on the following principles of treatment selection in these guidelines. When metastasis is found on imaging examinations, treatments such as radical surgery or chemotherapy are selected according to the extent of metastasis. When no evidence of metastasis is detected on imaging examinations, colorectal NETs with a low risk of metastasis are indicated for local resection, and those with a risk of metastasis are for radical surgery.

It is essential to evaluate the risk of metastasis from colorectal NETs to select a treatment for colorectal NETs without any metastasis findings on imaging examinations. Notably, previous studies that mainly examined surgically resected colorectal NETs have indicated that tumor size, invasion depth, NET grading based on mitotic index and/or Ki‐67 labeling index, and lymphovascular invasion are factors associated with metastasis.^4^, ^5^, ^6^, ^7^, ^8^, ^9^, ^10^, ^11^, ^12^ Because tumor size and depth can be estimated before treatment, these factors are used to select primary treatment, local resection, or radical surgery.

For invasion depth, it is widely accepted that the invasion to the muscularis propria or deeper is a vital risk factor for metastasis. Colorectal NETs with the invasion are indicated for radical surgery even though no metastasis findings are detected on imaging examinations.^4^, ^5^, ^6^, ^7^, ^8^, ^9^, ^10^, ^11^, ^12^ Regarding tumor size, a consensus has been reached that tumor size ≥20 mm is a risk factor for metastasis, and tumor size <10 mm is not.^4^, ^5^, ^6^, ^7^, ^8^, ^9^, ^10^, ^11^, ^12^ Therefore, every guideline proposes that colorectal NETs sized ≥20 mm are indicated for radical surgery, and those sized <10 mm without muscularis propria invasion are for local resection.^4^, ^5^, ^6^, ^7^ However, no consensus has been reached on the treatment strategy for colorectal NETs sized 10–19 mm without muscularis propria invasion. Several major international guidelines propose that local resection can be selected as the primary treatment for these lesions; in contrast, Japanese guidelines state that these lesions are indicated for radical surgery because of their undeniable risk of metastasis.^4^, ^5^, ^6^, ^7^ This discrepancy is confusing. Therefore, building a consensus on this issue is warranted by accumulating further evidence. This is particularly necessary for NETs located in the lower rectum, most frequently observed among colorectal NETs in Japan, because of their high invasiveness and influence on patients’ quality of life after radical surgery. Consequently, several studies have suggested the feasibility of diagnostic local resection for colorectal NETs up to 14 mm.^12^, ^13^, ^14^ A recent multicenter, retrospective study from South Korea assessed the clinical outcomes of 105 patients who underwent endoscopic resection for rectal NETs sized 10–20 mm (mainly 10–14 mm) and showed a good prognosis.^14^ This informative study may support the feasibility of endoscopic resection as a primary treatment for colorectal NETs of that size. However, this study's follow‐up period was not sufficiently long (the mean follow‐up period of 41.2 months), and three patients with metastasis were observed during this study period. Therefore, careful consideration is necessary, and further evidence is needed to address this issue.

## PROCEDURES OF ENDOSCOPIC RESECTION FOR COLORECTAL NET

Endoscopic resection has become the primary option for the local resection of colorectal NETs, although transanal surgery is another option for rectal NETs. Several endoscopic resection procedures are available, including polypectomy, conventional endoscopic mucosal resection (EMR), modified EMR (endoscopic submucosal resection with ligation device [ESMR‐L], EMR with a cap‐fitted panendoscope [EMR‐C], *etc*.), and endoscopic submucosal dissection (ESD).^7^, ^13^

Previously, polypectomy and conventional EMR were mainly selected for the endoscopic resection of colorectal NETs, resulting in a high proportion of a positive vertical resection margin.^13^, ^15^ This unfavorable result was caused by the fact that colorectal NETs are located mainly in the submucosa, a deeper site than the usual colorectal polyps and that polypectomy and conventional EMR cannot resect a sufficiently deep submucosal layer.

**FIGURE 1:**
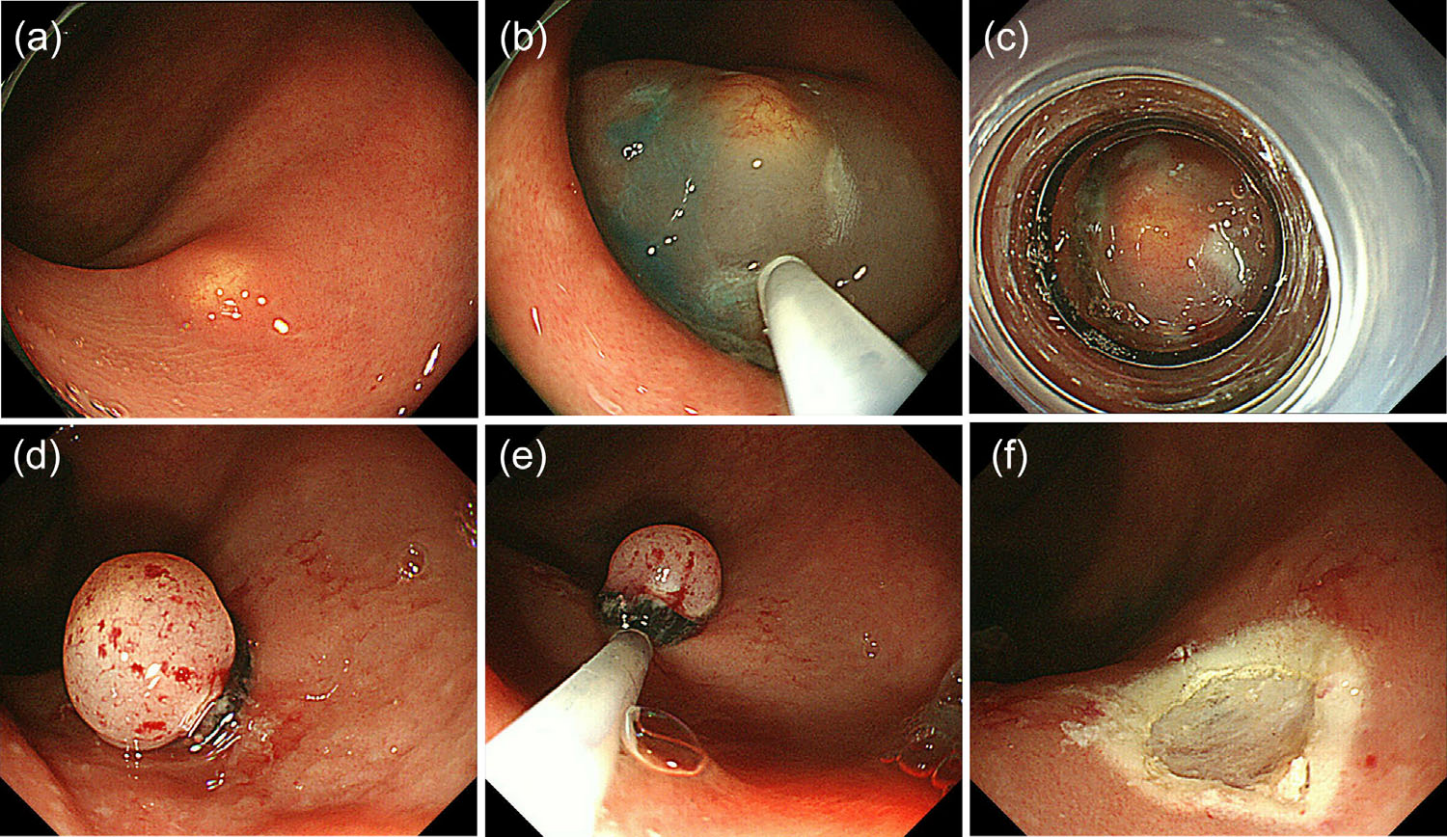
Endoscopic submucosal resection with ligation device for rectal neuroendocrine tumor: (a) a small neuroendocrine tumor in the lower rectum; (b) submucosal injection; (c) aspiration using a ligation device; (d) after releasing the band; (e) snaring under the band; (f) after resection.

Subsequently, ESMR‐L and EMR‐C were introduced for the endoscopic resection of colorectal NETs.^13^, ^15^, ^16^ A ligation device and transparent cap are used to aspirate the submucosal layer for ESMR‐L and EMR‐C, respectively, so that colorectal NETs can be entirely removed endoscopically with negative resection margins (Figure 1). A previous study from the National Cancer Center Hospital, Tokyo, vividly described the impact of introducing ESMR‐L for the treatment of rectal NETs despite the limited number of cases: The positive cut margin was found in 43% among rectal NETs before introducing ESMR‐L (between 1990 and 1997) but not found in any cases among rectal NETs after its introduction in 1999.^15^ Since then, more data have been accumulated on the clinical outcomes of ESMR‐L for colorectal NETs, and this procedure reportedly can provide >95% of R0 resection for colorectal NETs (mainly rectal NETs <10 mm).^13^, ^17^, ^18^, ^19^ It is also reported that EMR‐C can provide similarly high R0 resection rate for rectal NETs <10 mm.^13^, ^19^, ^20^ Notably, the simpler EMR‐C method, which does not require submucosal injection, has recently been proposed by a Chinese group, and its usefulness for rectal NET sized ≤10 mm has been demonstrated by a randomized noninferiority trial.^21^ They compared the simplified EMR‐C and ESD and proved the noninferiority of the EMR‐C compared with ESD in terms of complete resection rate (EMR‐C [38 cases] vs. ESD [41 cases]: 97.4% vs. 92.7%). In addition to effectiveness, the safety of ESMR‐L and EMR‐C is well known for treating colorectal NETs, particularly those located in the rectum lower than the peritoneal reflection, because of their little risk of perforation.^13^, ^15^, ^16^, ^17^, ^18^, ^19^, ^20^, ^21^ It is also worth noting that these procedures are convenient and easy to perform in an outpatient setting, requiring a short procedure time. In addition to ESMR‐L and EMR‐C, several other procedures have been developed as modified EMR techniques for colorectal NETs, including EMR with circumferential incision/precutting, anchored snare‐tip EMR, and underwater EMR, and their usefulness has been reported.^13^, ^19^, ^22^, ^23^, ^24^, ^25^

**FIGURE 2:**
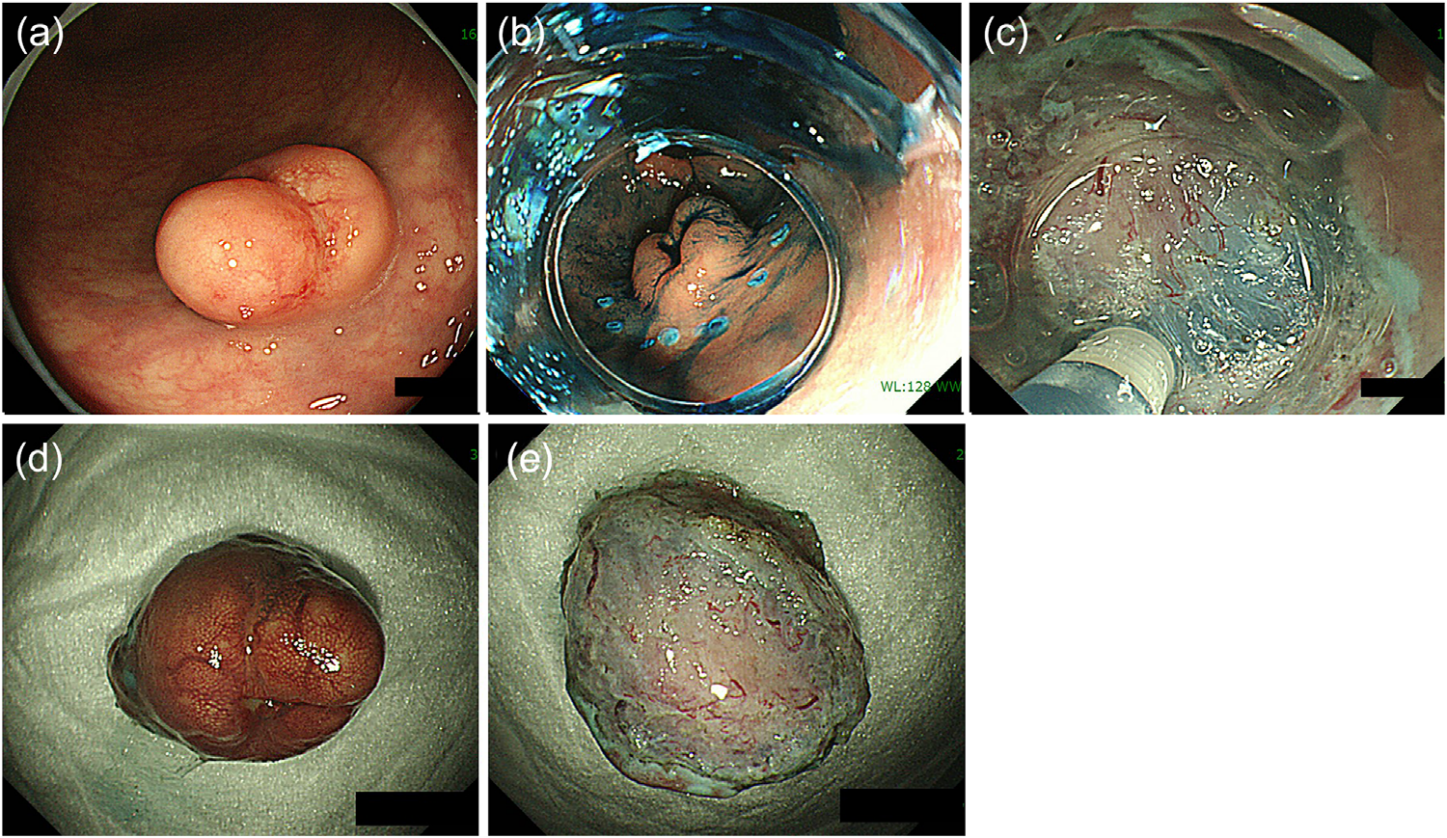
Diagnostic endoscopic submucosal dissection for rectal neuroendocrine tumor: (a) an intermediated‐sized neuroendocrine tumor in the lower rectum; (b) after marking and submucosal injection; (c) dissection just above the muscularis propria; (d,e) resected specimen.

Despite ESMR‐L and EMR‐C's usefulness, the effectiveness and safety of these procedures are not guaranteed when the lesion size is larger than can be aspirated into a suction device or when a lesion is located in the colon with a risk of perforation. In this case, ESD can be useful. ESD was initially developed for treating superficial GI cancer and has also been used for treating colorectal NETs. A high R0 resection rate is reportedly achieved for colorectal NETs with ESD; however, it is necessary to remember that dissection at a slightly superficial level can easily result in a positive vertical resection margin.^13^, ^19^ In fact, several studies have demonstrated the superiority of modified EMR to ESD in terms of R0 resection.^19^, ^26^, ^27^ Dissection at a sufficiently deep layer (just above the muscularis layer) is essential for achieving R0 resection for colorectal NETs with ESD (Figure 2).

In summary, several valuable options are available for the endoscopic resection of colorectal NETs. For rectal NETs <10 mm, modified EMRs such as ESMR‐L and EMR‐C seem favorable because of their ability of achieving a high R0 resection rate, safety, and convenience (short procedure time and no need for hospitalization). ESD can also be useful for treating these lesions but may be more effective for large or colon lesions.

## MANAGEMENT FOLLOWING LOCAL RESECTION OF COLORECTAL NET

Following the local resection of colorectal NETs, the pathological evaluation of the resected specimens was performed. The pathologically evaluated factors associated with metastasis are tumor size, invasion depth, tumor cell proliferative activity (NET grading), lymphovascular invasion, and resection margins.^7^ When colorectal NETs are judged to have a risk of metastasis based on the evaluation of these factors, radical surgery is recommended; otherwise, no treatment is recommended following endoscopic resection.^4^, ^5^, ^6^, ^7^

Tumor cell proliferative activity is a critical factor in deciding the management following local resection of colorectal NETs.^5^, ^6^, ^7^, ^28^ NET gradings are determined by assessing the tumor cell proliferative activity based on the mitotic index and/or Ki‐67 labeling index. Precise grading can be performed by evaluating whole resected lesions using a hotspot instead of biopsy specimens. Colorectal NETs are graded as one of NET G1, G2, and G3 based on the World Health Organization classification of NET grading (Table 1).^1^ NET G2 and G3 reportedly have a higher risk of metastasis than NET G1. Therefore, the Japanese guidelines recommend radical surgery for endoscopically resected colorectal NETs showing NET G2/3.^7^ The importance of NET grading is widely accepted; however, several other guidelines do not necessarily mention it in the context of subsequent treatment following local resection.^4^, ^5^, ^6^ In this situation, not a few physicians have difficulty determining whether radical surgery should be performed for colorectal NET G2 lesions, particularly rectal NET G2 lesions in which the Ki‐67 index is slightly over 3%.

**TABLE 1:** Grading of neuroendocrine tumor.^1^

NET grading	Differentiation	Ki‐67 index (%)	Mitotic count (mitoses/2 mm^2^)
NET G1	Well‐differentiated	<3	<2
NET G2		3–20	2–20
NET G3		>20	>20

Abbreviation: NET, neuroendocrine tumor.

Lymphovascular invasion is a hot topic in this field. Lymphovascular invasion, including lymphatic and venous invasion, was previously assessed only with hematoxylin and eosin staining. However, because tumor cells of colorectal NETs exhibit minute cytological atypia, the lymphovascular invasion of NETs is often difficult to identify. Even colorectal adenocarcinoma, which shows more remarkable cytological atypia than NET, is known to show interobserver variation in diagnosing lymphovascular invasion with hematoxylin and eosin staining, and the necessity of the special and immunohistochemical staining for a reproducible diagnosis has been recommended.^29^, ^30^, ^31^ The staining has recently been increasingly used to diagnose lymphovascular invasion even in colorectal NETs. Anti‐podoplanin (clone D2‐40) is used to detect lymphatic invasion, and Elastica van Gieson (EVG) staining and Victorian Blue staining are mainly used to detect venous invasion. Notably, with these stainings’ increased use, the proportion of positive lymphovascular invasion has risen remarkably, and positive lymphovascular invasion is frequently observed even in small colorectal NETs. This increase in the number of positive lymphovascular invasion in colorectal NETs has resulted in difficulty in deciding the appropriate management following local resection, particularly for small rectal NET G1 without invasion to the muscularis propria.

Previous studies that mainly examined surgically resected colorectal NETs have suggested that positive lymphovascular invasion is a strong risk factor for metastasis.^7^, ^8^, ^9^, ^10^, ^11^, ^12^ Therefore, the Japanese guidelines state that colorectal NETs with positive lymphovascular invasion are indicated for radical surgery following local resection.^7^, ^8^, ^9^, ^10^, ^11^, ^12^ However, few studies have systematically examined lymphovascular invasion using the immunohistochemical and special staining; therefore, it is unknown whether the obtained findings on the clinical significance of lymphovascular invasion from these previous studies can be applied to cases with positive lymphovascular invasion, which is detected using the recent immunohistochemical and/or special staining. In this context, a previous study from National Cancer Center Hospital, Tokyo has provided informative findings on this issue.^18^ In this study, lymphovascular invasion in 90 rectal NETs in 86 patients treated with endoscopic resection between January 1997 and December 2011 was reevaluated by newly using the special and immunohistochemical staining. For the accurate detection of lymphovascular invasion, in addition to EVG staining, the double‐staining immunohistochemistry for endothelial and neuroendocrine markers was used. Concretely, anti‐synaptophysin antibody was used in combination with anti‐podoplanin and anti‐CD31 antibody. As a result, although these colorectal NETs were all NET G1 without invasion to the muscularis propria and the median tumor size was 5 mm, the proportion of positive lymphovascular invasion turned out to be surprisingly high (46.7%; positive lymphatic invasion: 25.6%; positive venous invasion: 36.7%). In addition, these patients did not show any recurrence without secondary treatment after endoscopic resection during the median follow‐up period of >5 years. These findings raise questions regarding the clinical significance of lymphovascular invasion in small rectal NETs detected using the staining. Since this study's publication, several other retrospective studies from different institutions have reported similar results, emphasizing the importance of this issue.^32^, ^33^, ^34^, ^35^, ^36^ Furthermore, a recent Japanese multicenter prospective study on colorectal NETs, the details described later, has proven high positivity of lymphovascular invasion in colorectal NETs based on the assessment of prospectively enrolled colorectal NET patients.^37^ There is a potential that follow‐up without secondary treatment for colorectal NETs with positive lymphovascular invasion can be a feasible management option when they do not have any other metastatic risk factors; however, more evidence is necessary.

**FIGURE 3:**
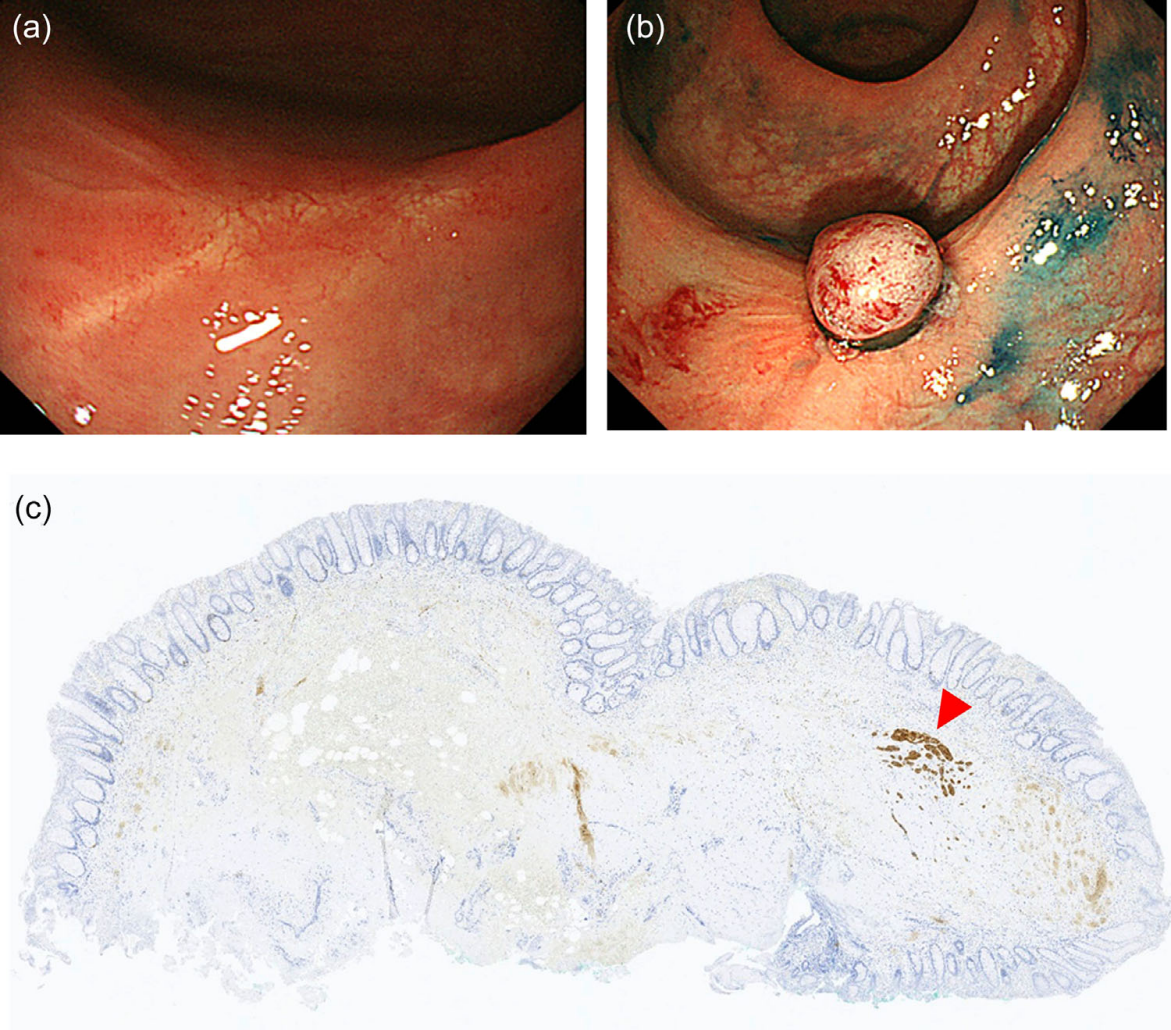
Endoscopic submucosal resection with ligation device for a scar by previous endoscopic resection for rectal neuroendocrine tumor with a positive resection margin: (a) a scar by previous endoscopic resection for rectal neuroendocrine tumor with a positive resection margin; (b) endoscopic submucosal resection with ligation device performed for the scar; (c) pathological evaluation (anti‐synaptophysin antibody) of the resected specimen showing a diminutive component of the neuroendocrine tumor (red arrowhead).

Concerning resection margins, the Japanese guidelines recommend radical surgery following endoscopic resection with a positive resection margin.^7^ In particular, a positive vertical resection margin is regarded as the status requiring surgery. However, due to rectal surgery's high invasiveness, deciding to perform surgery for rectal NETs with positive resection margins is often difficult in real‐world practice. A multicenter retrospective study from South Korea showed a low recurrence rate (1.5% [2/134]) among rectal NET patients who did not undergo secondary treatment following endoscopic resection with indeterminate or positive resection margins of endoscopic resection for rectal NETs, suggesting that invasive radical surgery is not necessarily required for cases with a positive resection margin.^38^ However, the median follow‐up period was shorter than 5 years in the study, and longer follow‐up may increase recurrence. In this context, instead of follow‐up without any treatment, secondary endoscopic resection may be another management option following a positive resection margin, despite the lack of supporting evidence. Modified EMR and ESD may be used to perform endoscopic resection of scars, and pathological evaluation of the resected specimen may help determine further management. A residual component of colorectal NET can be infrequently observed in specimens that undergo secondary endoscopic resection (Figure 3).

As described above, confusion remains on the management following local resection with NET grading ≥2, positive lymphovascular invasion, and positive resection margins. Further investigation and discussion are necessary to reach a consensus. Since the significance of lymph node metastasis, particularly micrometastasis from small colorectal NETs, as a prognostic factor has not yet been established, more evidence based on long‐term clinical outcomes is warranted. Data on the long‐term clinical outcomes of colorectal NET patients with a sufficiently long follow‐up period and consideration of all important lesion factors (not only size and depth but also NET grading, lymphovascular invasion, and resection margins) are presently lacking. Therefore, appropriate follow‐up periods are unknown and surveillance programs are yet to be established.

## C‐NET STUDY

To achieve high‐quality data on the long‐term clinical outcomes of colorectal NET patients and contribute to the establishment of their appropriate treatment and follow‐up, a multicenter prospective study of colorectal NETs, the “C‐NET study” (UMIN‐CTR number, UMIN000025215), is ongoing in Japan.^37^ This study prospectively enrolled colorectal NET patients at 50 participating institutions and currently continues a 10‐year‐follow‐up to assess their long‐term outcomes. It started in January 2017, and enrollment ended in December 2019; a total of 495 patients with 500 colorectal NETs were included. Consequently, more time is required to obtain long‐term outcomes; however, the characteristics of the enrolled patients and lesions have already been reported in their first paper.^18^ Most colorectal NETs were incidentally found in asymptomatic patients of the working generation (median age, 54 years) with colorectal cancer screening. Most lesions were small NET G1 without invasion to the muscularis propria and were located in the lower rectum. Nevertheless, the proportion of patients with positive lymphovascular invasion was high. With the immunohistochemical and special staining, the proportion of positive lymphovascular invasion was remarkably high, even in small NETs: 26.4% and 40.9% in lesions <5 and 5–9 mm, respectively. It is meaningful that such a high positivity rate was proven in a prospective study. In the future, C‐NET study will provide informative findings regarding the clinical significance of positive lymphovascular invasion based on long‐term outcomes.

## CONCLUSIONS

This review summarizes the current status of the treatment and post‐treatment management of colorectal NETs. Despite the development in treatment and pathological evaluation for colorectal NETs, there are still issues on which non‐consensus has been reached, including the appropriate treatment for colorectal NETs sized 10–19 mm, and management following local resection of lesions with NET grading ≥2, positive lymphovascular invasion, and positive resection margins. Further data on the long‐term clinical outcomes of colorectal NETs are required to address these issues.

## CONFLICT OF INTEREST STATEMENT

The authors have no conflict of interest.
